# Identification of the first congenital ichthyosis case caused by a homozygous deletion in the *ALOX12B* gene due to chromosome 17 mixed uniparental disomy

**DOI:** 10.3389/fgene.2022.931833

**Published:** 2022-08-08

**Authors:** Lei Zhang, Yanqiu Hu, Jingjing Lu, Peiwei Zhao, Xiankai Zhang, Li Tan, Jun Li, Cuiping Xiao, Linkong Zeng, Xuelian He

**Affiliations:** ^1^ Precision Medical Center, Wuhan Children’s Hospital (Wuhan Maternal and Child Healthcare Hospital), Tongji Medical College, Huazhong University of Science & Technology, Wuhan, China; ^2^ Dermatology Department, Wuhan Children’s Hospital (Wuhan Maternal and Child Healthcare Hospital), Tongji Medical College, Huazhong University of Science & Technology, Wuhan, China; ^3^ Otolaryngology Department, Wuhan Children’s Hospital (Wuhan Maternal and Child Healthcare Hospital), Tongji Medical College, Huazhong University of Science & Technology, Wuhan, China; ^4^ Neonatology Department, Wuhan Children’s Hospital (Wuhan Maternal and Child Healthcare Hospital), Tongji Medical College, Huazhong University of Science & Technology, Wuhan, China

**Keywords:** ARCI, *ALOX12B*, whole-exome sequencing, mixed UPD (mixUPD), microtia

## Abstract

Uniparental disomy (UPD) is a rare genetic event caused by errors during gametogenesis and fertilization leading to two copies of a chromosome or chromosomal region inherited from one parent. MixUPD is one type of UPD that contains isodisomic and heterodisomic parts because of meiotic recombination. Using whole-exome sequencing (WES), we identified the first case of ichthyosis due to a maternal mixUPD on chromosome 17, which results in a homozygous deletion of partial intron 8 to exon 10 in *ALOX12B*, being predicted to lead to an internal protein deletion of 97 amino acids. We also performed a retrospective analysis of 198 patients with *ALOX12B* mutations. The results suggested that the exon 9 and 10 are located in the mutational hotspots of *ALOX12B*. In addition, our patient has microtia and congenital stenosis of the external auditory canals, which is very rare in patients with *ALOX12B* mutations. Our study reports the first case of autosomal recessive congenital ichthyosis (ARCI) due to a mixUPD of chromosome 17 and expands the spectrum of clinical manifestations of ARCI caused by mutations in the *ALOX12B* gene.

## Introduction

Uniparental disomy (UPD), first reported by Engel in 1980, refers to the inheritance of two copies of chromosomes or their segments from one parent, different from the classical Mendelian inheritance (Engel, 1980). The two copies can be identical (uniparental isodisomy or iUPD), in which a single chromosome from one parent is duplicated (caused by meiosis II error), or not identical (uniparental heterodisomy or hUPD), in which a pair of nonidentical chromosomes are inherited from one parent (caused by a meiosis I error) (Liehr, 2010). If a UPD contains iUPD and hUPD, with either terminal region of homozygosity (ROH) (meiosis I error) or centromeric ROH (meiosis II error), it is referred to as a mixed UPD (mixUPD) due to meiotic recombination (Yauy et al., 2020). UPD can result in imprinting disorders when it involves chromosomes 6, 7, 11, 14, 15, or 20 (Scuffins et al., 2021), such as Prader-Willi/Angelman syndrome in 15q11-q13 (Sharp et al., 2010). In addition, UPD is a cause of autosomal recessive (AR) disorders through the inheritance of recessive disease alleles from a carrier parent, which only occurs in iUPD or mixUPD patients (Kotzot, 2001; Spiekerkoetter et al., 2002; King et al., 2014; Xiao et al., 2019).

Autosomal recessive congenital ichthyosis (ARCI) is a group of genetic diseases related to defective epidermal barriers; to date, 13 known genes have been causally associated with this disorder, namely, *ALOX12B*, *ABCA12*, *ALOXE3*, *CERS3*, *CYP4F22*, *LIPN*, *NIPAL4*, *PNPLA1*, *SDR9C7*, *SLC27A4*, *SULT2B1*, *ST14*, and *TGM1* (Williams and Elias, 1985; Richard, 1993; Vahlquist et al., 2018; Simpson et al., 2020). The *ALOX12B* gene encodes the keratinocyte lipid transporter ATP-binding cassette subfamily A, and mutations in this gene, which lead to the disruption of lamellar granule lipid transport in the upper epidermal keratinocytes, cause nonbullous congenital ichthyosiform erythroderma (NCIE, MIM: #242100) or mild phenotypes such as self-improving collodion ichthyosis (SICI) (Jobard et al., 2002; Harting et al., 2008; Vahlquist et al., 2010). In addition to skin symptoms, overfolded ear and hearing loss have been presented in cases with *ALOX12B* mutations in a recent study (Simpson et al., 2020).

Here, we report the first case with SICI caused by homozygous deletion of the entire exon 9 and partial exon 10 in the *ALOX12B* gene, and this deletion has induced exon 9 and 10 skipping, likely resulting in a mutation protein with an internal absence of 97 amino acids. Further homozygosity mapping and B allele frequency (BAF) analysis have indicated that the homozygous deletion of *ALOX12B* was caused by a maternal mixUPD of chromosome 17. In addition to SICI, our patient had congenital auricular deformities, microtia, and stenosis of the external auditory canals.

## Case presentation

### Clinical report

**FIGURE 1:**
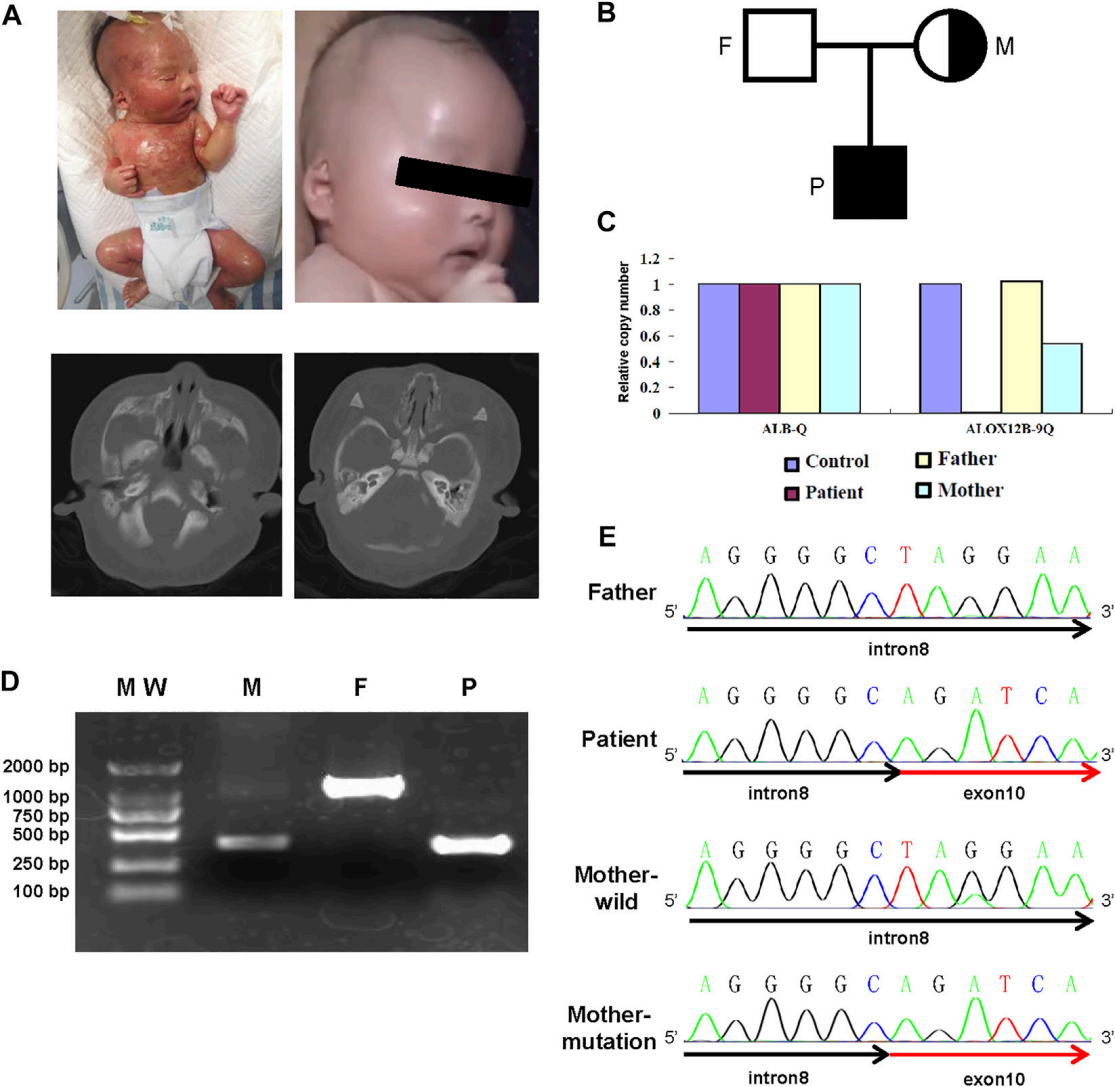
Clinical and genetic information of the family. **(A)** Photograph and inner ear CT of the case. Lateral view of the patient at the age of 1 day (upper left panel) and 3 months (upper right panel). CT plain scan of the inner ear (lower panel). **(B)** Pedigree of the reported family. **(C)** Quantitative PCR histogram of *ALOX12B* to *ALB gene.* **(D)** Agarose gel electrophoresis of PCR amplification for samples from the patient (P), heterozygous carrier mother (M), and wild-type father (F). **(E)** Sanger sequencing confirms the breakpoints of the deletion encompassing the 3′ end of intron 8 and 5′ end of exon 10.

The newborn boy was the second child of healthy non-consanguineous parents without skin disorders (Figure 1B). He was delivered by cesarean section at 39 weeks of gestation after an uneventful pregnancy. His birth weight was 3,060 g, and his height was 50 cm with an Apgar score of 9 at 1 minute and 9 at 5 minutes. At birth, he was covered by a collodion membrane with underlying erythroderma. His skin was covered with multiple fissures, together with partial desquamation and oozing through its cracks. He had bilateral ectropion and eyelid swelling. In addition, he had a significant malformation of the external ear and microtia, and further CT scanning of the inner ear showed that the cartilaginous part was narrow (left side) (Figure 1A). Left inguinal canal cryptorchidism was noted.

The patient was immediately admitted to the intensive care unit and given liver oil ointment to the entire body to keep the affected area moist and mupirocin ointment to reduce secondary bacterial infection where the skin was broken. Erythromycin ophthalmic ointment was applied on the eyelid margins to reduce eyelid swelling. After 2 weeks of treatment, the membrane was shed, and the skin looked almost normal after a 3-month follow-up (Figure 1A). In addition, he had no significant hearing problems.

## Methods

The study was approved by the Clinical Research Ethics Committee of Wuhan Children’s Hospital, Tongji Medical College, Huazhong University of Science and Technology, and written informed consent was obtained from the patient’s parents.

### Whole-exome sequencing and homozygosity mapping analysis

Genomic DNA was isolated from the peripheral blood, and trio-WES was conducted on the proband and his parents. The protocol used for WES was the same as previously described (Luo et al., 2021). WES data analysis showed a loss of heterozygosity (LOH) region of 15.33 Mb in the short arm of chromosome 17. To further confirm the LOH, the clean data were aligned to the human reference genome (hg19) using BWA-MEM (v.0.7.12), then sorted and indexed with SAMtools (version 1.3.1). Single-nucleotide polymorphism (SNP) calling and mapping were performed using the GATK (version 3.8) Haplotype Caller. After common CNV (>1% population frequency) regions were filtered out with the aid of PLINK(R), SNPs were selected based on the population databases (dbSNP153) with minor allele frequency values between 20 and 80%.

Genotypic status at each locus was assessed as BAF, the locus with a value of 0.5 is the heterozygous position and 1 or 0 is for the homozygous genotype. By estimating the proportion of homozygous/heterozygous genotypes for all candidate loci in each chromosome, a preliminary judgment was made on whether partial or whole chromosome UPD was present. For the sliding window homozygosity analysis, the window size was set to 50 SNPs, and the sliding stride was set to 5 SNPs with *r* ^2^ > 0.5 in 2 SNPs. A fragment of homozygous region (ROH) was defined as follows: a segment ≥2.5 Mb in length contains over 50 homozygous SNPs distributed with a density of at least 1 SNP per 5,000 kb.

### Quantitative polymerase chain reaction of *ALOX12B*

The genomic DNA was adjusted to 20 ng/μL using the Qubit 2.0 fluorometer and Qubit dsDNA HS Assay kit. Quantitative PCR was performed using the quantitative TAKARA SYBR Green PCR kit and ABI StepOnePlus PCR instrument according to the following conditions: 95°C for 2 min, followed by 95°C for 15 s, and 60°C for 60 s (40 cycles). We quantified ALB gene (encoding albumin, 4q13.3) as the internal reference gene. The primers for these DNA fragments were ALB exon 13 (forward: 5′-AGT GCA CTT GTT GAG CTC GTG-3′, reverse: 5′-GCA AAG CAG GTC TCC TTA TCG-3′), *ALOX12B* exon 9 (forward: 5′-GCC ACC CCC TCT ACA AGG TAT-3′, reverse: 5′-TAT GCC AAG CCC TCT CTT GTG-3′).

### Deletion breakpoint analysis of the *ALOX12B* gene

The WES analysis suggested that the proband carried a deletion of exon 9 in the *ALOX12B* gene. To determine the breakpoint, primers were designed from the breakpoint interval, with the upstream primer being complementary to sequences in intron 8, and the downstream primer being complementary to sequences in intron 10 (forward: 5′-GTC AGG TCC CTA CTG GAA ATAC-3′, reverse: 5′-ATG ATG ACC CAA AGG CAA A-3′). A long-range PCR was performed using FastPfu DNA Polymerase (TransGen Biotech), and the products were separated on 1.5% agarose gels and sequenced by the ABI 3500 DNA Sequencer (Applied Biosystems, United States).

### Review of reported *ALOX12B* cases from literature

The databases such as PubMed, Scopus, Embase, and Web of Science (including MEDLINE) were systematically searched through 2022/02. The search terms included the combination of the following groups of keywords: 1) “*ALOX12B*gene,” or “Arachidonate 12-Lipoxygenase”; 2) “variant” or “mutation”; and 3) “recessive congenital ichthyosis” or “self-improving collodion ichthyosis.” The initial electronic search identified 90 articles in PubMed, 46 in Scopus, 92 in Embase, and 77 in Web of Science. After removing the duplicates and screening the articles by title and abstract, we discarded 281 articles, and 24 remained (Jobard et al., 2002; Eckl et al., 2005; Ashoor et al., 2006; Lesueur et al., 2007; Harting et al., 2008; Eckl et al., 2009; Akiyama et al., 2010; Kurban et al., 2010; Vahlquist et al., 2010; Li et al., 2012; Chaitidis et al., 2013; Israeli et al., 2013; Osorio et al., 2013; Numata et al., 2015; Lolas et al., 2016; Bastaki et al., 2017; Alavi et al., 2020; Fioretti et al., 2020; Anker et al., 2021; Frommherz et al., 2021; Hotz et al., 2021; Mohamad et al., 2021; Srinivas et al., 2021). Mutations in the *ALOX12B* gene were described according to the Human Genome Variation Society, using the NCBI reference sequence NM_001139.3. After completing the statistical analysis, a lollipop diagram was created by the trackViewer (version 1.28.1) in R software (version 3.6.3). Density plots were generated with ggplot2, and “geom_density” function was used to analyze the possible mutation spectrum and hotspots.

## Results

### Trio-whole-exome sequencing data analysis reveals a reduction in homozygosity for a maternal *ALOX12B* deletion mutation due to maternal mixUPD

**FIGURE 2:**
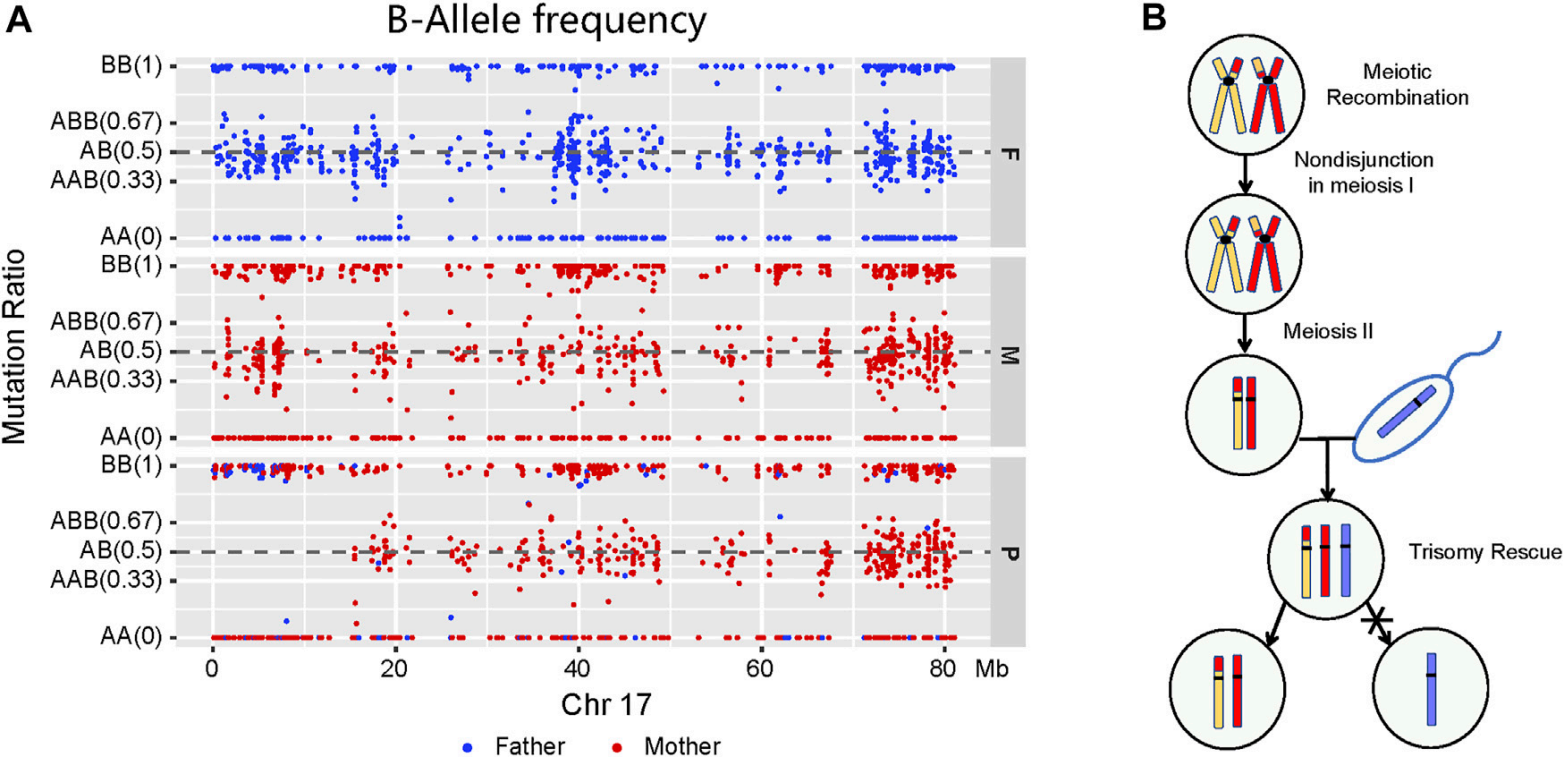
Molecular characterization and a model for the origin of mixUPD. **(A)** A loss of LOH region in the short arm of chromosome 17 described by B-allele frequency plot. Paternal and maternal sites are highlighted in blue and red, respectively. A point is classified as AA—if the BAF value is <0.1, as AB/AAB/ABB—if it is between 0.1 and 0.9, and as BB if it is >0.9. LOH takes place on the short arm of chromosome 17 (p11.2–p13.3) in the patient, and most points are generated maternally. **(B)** Schematic illustration of genetic events with mixUPD of chromosome 17.

Trio-WES and homozygosity mapping detected the LOH region of 15.33 Mb in the short arm of chromosome 17 (17p), which included the *ALOX12B* gene. To further investigate the LOH occurrence on chromosome 17, BAF was performed by checking Mendelian inheritance errors in the trio-WES data. A total of 1,286 SNPs, ranging 80 Mb on chromosome 17, were analyzed, and 1,119 (87.0%) SNPs, which included homozygous and heterozygous SNPs, were identified as being maternally inherited, suggesting a maternal mixUPD in the patient (Figure 2A). Thus, the homozygous deletion of *ALOX12B* exon 9 was caused by a maternal isodisomy. Figure 2B explains the mechanisms of chromosome 17 mixUPD caused by errors in stages I and II of meiosis.

### Validation of the partial *ALOX12B* gene deletion and breakpoint analysis

The partial deletion of *ALOX12B* gene was validated by quantitative PCR analysis, and the results revealed that the ratios in exon 9 regions were 0, 0.5, and 1 in the proband, his mother, and his father, respectively (Figure 1C). PCR using primers complementary to sequences in introns 8 and 10 revealed that the proband was homozygous for deletion, whereas his mother was heterozygous and his father was normal (Figure 1D). Sanger sequencing was used to detect the breakpoint, and alignment to hg19 revealed the breakpoints located within IVS8 at the g.8077544_8077545 position and the distal one within exon10 at the g.8076713_8076714 position, resulting in 831 bp deletion, which included the full length of exon 9 and intron 9 and partial exon 10 (Figure 1E).

### Review of *ALOX12B* mutations in self-improving collodion ichthyosis

**FIGURE 3:**
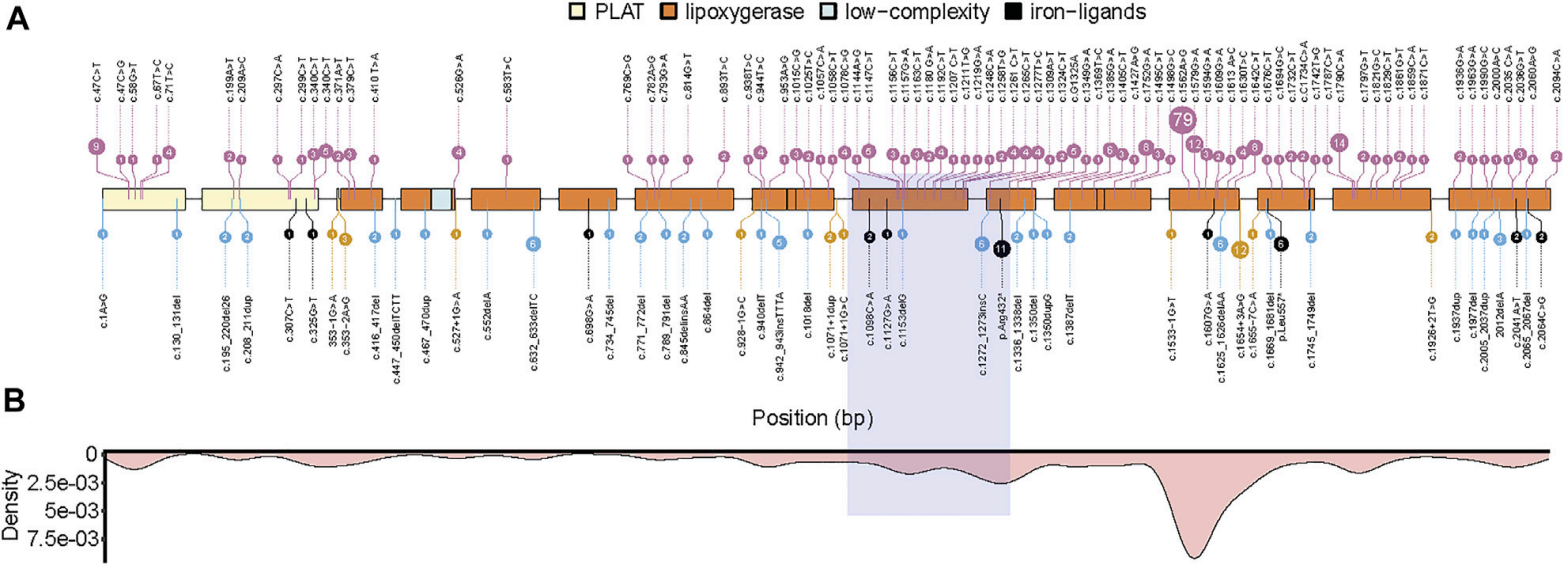
Summary of pathogenic mutations in *ALOX12B*. **(A)** *ALOX12B* domains, exons, and site of variants (missense mutations in pink, frameshift mutations in blue, nonsense mutations in black, and splicing mutations in yellow). **(B)** The density plot of all mutations is shown below, and the light purple box represents the deletion in this case. PLAT: polycystin-1, lipoxygenase, α-toxin domain.

We reviewed and listed 134 different pathologic mutation sites in the *ALOX12B* gene reported in 198 ARCI patients (Figure 3A). Among these, missense and frameshift mutations were the most common types, accounting for more than 80% of the cases. The high-frequency mutation sites were more concentrated in exons 9,10,12, and 14, which are located in the lipoxygenase domain (Figure 3B). The homozygous mutation in our case is in the hotspot region and presents with SICI.

## Discussion

We report the first case of ARCI resulting from a homozygous deletion of the 3’ end of intron 8, exon 9, intron 9, and part of exon 10 (831 bp) in the *ALOX12B* gene due to a mixUPD of maternal chromosome 17. In addition to ichthyosis, our patient had microtia and congenital stenosis of the external auditory canals, which is exceedingly rare in patients with gene mutations.

UPD is an underestimated cause of AR disorders. Previously, the most cited prevalence of UPD was 1/3,500 live births (Robinson, 2000). However, recent studies have estimated that UPD occurs 1 in 2,000 births, and maternal UPD is twice as prevalent as paternal UPD (Nakka et al., 2019; Scuffins et al., 2021). Chromosome 17 UPD is uncommon, and the first case of maternal UPD involving chromosome 17 was reported in a child with a normal phenotype in 1999 (Genuardi et al., 1999). Although UPD of chromosome 17 is not associated with imprinting disease, it has the potential to unmask recessive mutations. To date, only a few cases with genetic conditions caused by UPD of chromosome 17 have been reported (Lebre et al., 2009; Natsuga et al., 2010; Labrijn-Marks et al., 2019).

ARCI is a rare dermatological condition, and to date, mutations in 13 genes have been identified to cause it (Williams and Elias, 1985; Richard, 1993; Vahlquist et al., 2018). However, there are only five cases of ichthyosis caused by UPD involving *CERS3* (2), *ABCA12* (2), and *SPINK*5 (1) (Castiglia et al., 2009; Numata et al., 2014; Shibata et al., 2015; Polubothu et al., 2018; Muthusamy et al., 2020). To our knowledge, no case involving UPD of chromosome 17 has been reported to have ichthyosis, and our patient is the first case with ARCI caused by UPD.

Interestingly, we reported that the baby showed congenital auricular deformities. The phenomenon was not reported in a large cohort until nearly 2 years ago and the total number does not exceed 10 (Simpson et al., 2020; Hotz et al., 2021). However, the location and types of mutations on *ALOX12B* do not seem to affect the severity of dysplastic ears (Simpson et al., 2020), and the mechanism by which *ALOX12B* mutations cause dysplastic ears is definitively unknown. Furthermore, in this case, the proband presented with cryptorchidism, which has not been reported yet to be associated with the *ALOX12B* gene.

By literature review, we found 134 different pathologic mutations reported in the *ALOX12B* gene among 198 ARCI patients, with missense mutation being the most frequent type, followed by frameshift mutations. The hotspot regions are exons 9, 10, 12, and 14, which encode the lipoxygenase domain (Figures 3A,B). The deletion in our case was due to UPD and was in the hotspot region, exon 9, and part of exon 10. To date, neither homozygous deletion nor mutation due to UPD has been reported. In this study, a homozygous deletion of the 3’ end of intron 8, exon 9, and part of exon 10 was predicted to cause a shortened ALOX12B production (in-frame transcripts) or degradation of aberrant ALOX12B mRNA induced by the nonsense-mediated decay, both of which would cause the loss of function of ALOX12B. It has been previously reported that a 35-year-old male with homozygous deletion of ALOX12B exon 3–15 had a mild body erythema in his childhood and only very mild face erythema with palmar hyperlinearity in adulthood (Fioretti et al., 2020). It is unlikely that the mutant ALOX12B genes with deletion of exon 3–15 can produce a protein with residual enzyme activity, thus we speculate that ALOX12B deficiency alone causes a mild, self-improving, ARCI phenotype, and that more severe ALOX12B-related phenotypes could depend on additional modifiers, genetic or epigenetic ones. Our patient with the homozygous deletion of partial intron 8 to exon 10 in *ALOX12B* also displayed relative mild skin phenotypes, and the deletion is predicted to cause the in-frame skipping of exon 9 and 10, with exon 8 joined to exon 11, resulting in an internal protein deletion of 97 amino acids (from Leu358 to Lys454), suggesting that this region is not critical for enzyme activity.

Lastly, our study supports that the WES data can identify distinct types of genomic lesions, such as SNVs, LOH, and CNVs (Xiao et al., 2019). Detecting UPD events in the trio-WES data does not incur additional costs but increases the diagnostic rate, especially in cases with non-Mendelian inheritance (Xiao et al., 2019; Yauy et al., 2020).

## Conclusion

Here, we report the first case of ARCI due to homozygous deletion of exon 9 and partial exon 10 in the *ALOX12B* gene due to mixUPD of maternal chromosome 17. Our study extends the clinical phenotype spectrum and further confirms that congenital auricular deformity is one of the phenotypes in patients with *ALOX12B* gene mutations. Furthermore, our work supports the value that trio-WES can have as an auxiliary diagnostic method for testing UPD.

## Data Availability

The datasets for this article are not publicly available due to concerns regarding participant/patient anonymity. Requests to access the datasets should be directed to the corresponding authors.
