# Corrigendum: Identification of the first congenital ichthyosis case caused by a homozygous deletion in the *ALOX12B* gene due to chromosome 17 mixed uniparental disomy

**DOI:** 10.3389/fgene.2022.1036144

**Published:** 2022-10-21

**Authors:** Lei Zhang, Yanqiu Hu, Jingjing Lu, Peiwei Zhao, Xiankai Zhang, Li Tan, Jun Li, Cuiping Xiao, Linkong Zeng, Xuelian He

**Affiliations:** ^1^ Precision Medical Center, Wuhan Children’s Hospital (Wuhan Maternal and Child Healthcare Hospital), Tongji Medical College, Huazhong University of Science & Technology, Wuhan, China; ^2^ Dermatology Department, Wuhan Children’s Hospital (Wuhan Maternal and Child Healthcare Hospital), Tongji Medical College, Huazhong University of Science & Technology, Wuhan, China; ^3^ Otolaryngology Department, Wuhan Children’s Hospital (Wuhan Maternal and Child Healthcare Hospital), Tongji Medical College, Huazhong University of Science & Technology, Wuhan, China; ^4^ Neonatology Department, Wuhan Children’s Hospital (Wuhan Maternal and Child Healthcare Hospital), Tongji Medical College, Huazhong University of Science & Technology, Wuhan, China

**Keywords:** ARCI, *ALOX12B*, whole-exome sequencing, mixed UPD (mixUPD), microtia

In the published article, there was an error in affiliations 1, 2, and 3.

In all three affiliations, “Huazhong University of Science & Technology, Wuhan, China”, should be “Wuhan Children’s Hospital (Wuhan Maternal and Child Healthcare), Tongji Medical College, Huazhong University of Science & Technology, Wuhan, China”.

The authors apologize for this error and state that this does not change the scientific conclusions of the article in any way. The original article has been updated.

